# Regulation of sister chromatid cohesion by nuclear PD-L1

**DOI:** 10.1038/s41422-020-0315-8

**Published:** 2020-04-29

**Authors:** Jia Yu, Bo Qin, Ann M. Moyer, Somaira Nowsheen, Xinyi Tu, Haidong Dong, Judy C. Boughey, Matthew P. Goetz, Richard Weinshilboum, Zhenkun Lou, Liewei Wang

**Affiliations:** 1 Department of Molecular Pharmacology and Experimental Therapeutics, Mayo Clinic, Rochester, MN 55905 USA; 2 Department of Oncology, Mayo Clinic, Rochester, MN 55905 USA; 3 Department of Laboratory Medicine and Pathology, Mayo Clinic, Rochester, MN 55905 USA; 4 Mayo Clinic Graduate School of Biomedical Sciences, Mayo Clinic School of Medicine and the Mayo Clinic Medical Scientist Training Program, Mayo Clinic, Rochester, MN 55905 USA; 5 Departments of Urology and Immunology, Mayo Clinic, Rochester, MN 55905 USA; 6 Department of Surgery, Mayo Clinic, Rochester, MN 55905 USA

**Keywords:** Oncogenes, Cell division

## Abstract

Programmed death ligand-1 (PD-L1 or B7-H1) is well known for its role in immune checkpoint regulation, but its function inside the tumor cells has rarely been explored. Here we report that nuclear PD-L1 is important for cancer cell sister chromatid cohesion. We found that depletion of PD-L1 suppresses cancer cell proliferation, colony formation in vitro, and tumor growth in vivo in immune-deficient NSG mice independent of its role in immune checkpoint. Specifically, PD-L1 functions as a subunit of the cohesin complex, and its deficiency leads to formation of multinucleated cells and causes a defect in sister chromatid cohesion. Mechanistically, PD-L1 compensates for the loss of Sororin, whose expression is suppressed in cancer cells overexpressing PD-L1. PD-L1 competes with Wing Apart-Like (WAPL) for binding to PDS5B, and secures proper sister chromatid cohesion and segregation. Our findings suggest an important role for nuclear PD-L1 in cancer cells independent of its function in immune checkpoint.

## Introduction

Immune checkpoints are cell surface receptors that are expressed in immune cells. They modulate the amplitude and quality of the adaptive and innate effectors, thus maintaining immune homeostasis and preventing autoimmunity.^1^ Well-studied immune checkpoint proteins include cytotoxic T-lymphocyte protein 4 (CTLA4) and programmed cell death protein 1 (PDCD1; also known as PD1). CTLA4 pathways restrict T cell activity at the early stage of immune response, whereas PD1 signaling plays roles in the later stage of immune response, protecting surrounding tissues at sites of chronic inflammation from damage.^2^ Both immune checkpoints are utilized by cancer cells to evade immune surveillance. PD1, also known as PDCD1 or CD279, is a membrane protein and expressed on T cells and pro-B cells.^3–5^ Its ligand, PD-L1, also referred to as B7-H1 or CD274, is a type 1 transmembrane protein that binds to PD1 and negatively regulates T cell function and survival.^6^ The expression of PD-L1 in the tumor microenvironment protects cancer from immune-mediated rejection.^7^ PD-L1 is up-regulated in a variety of human carcinomas, including breast cancer, ovarian cancer, colon cancer, melanoma and lung cancer.^7,8^ PD-L1 assists tumor cells evade the host immune system. Consequently, several checkpoint inhibitors, such as anti-PD-L1 monoclonal antibody, have been developed to block this inhibitory pathway and reactivate T-cell activity against cancer cells.^6,9^ High levels of PD-1 expression are detected in circulating and tumor-infiltrating lymphocytes in different cancer tissues.^10–13^ These antibodies function by blocking tumor PD-L1-PD-1 interaction that preserves the PD-1 expressing anti-tumor T cell function, so that they can attack tumor cells expressing PD-L1.

Due to the huge success of anti-PD-L1 and anti-PD-1 therapy, much effort has been put into studying the interaction between the tumor PD-L1 and T cell PD1 in cancer immunotherapy. Recently, only a few studies have explored the functions of PD-L1 inside tumor cells. These studies suggest that tumor PD-L1 suppresses tumor apoptosis, modulates chemo-resistance through MAPK/ERK activation, controls tumor glucose metabolism and regulates autophagy in ovarian cancer and melanoma.^14–16^ Interestingly, nuclear staining of PD-L1 is detected in cancer tissues.^17^ However, the function of nuclear PD-L1 in cancer cells is rarely explored.

PD-L1 is overexpressed in ~20% of triple negative breast cancers (TNBCs),^18^ a subtype of breast cancer with a very poor prognosis. Recent phase I studies using antibodies that target PD-L1 (atezolizumab) in women with metastatic TNBC have demonstrated that a minority of women (<10%) exhibit tumor response.^19,20^ These studies prompted us to use TNBC as one of the models to study PD-L1 function in tumor.

Cohesin is a highly conserved chromosome-associated multi-subunit protein complex in eukaryotes. It is composed of four core subunits-SMC1, SMC3, SCC1 (RAD21), and either SA1 (STAG1) or SA2 (STAG2) and three regulatory subunits-PDS5B, WAPL and Sororin.^21–28^ The cohesin core subunits form a ring-shaped structure. SMC1 and SMC3 dimerize with hinge domain and their other ends are bridged by SCC1. SA1/2 and PDS5B associate with SCC1. Through PDS5B, the sub-stoichiometric regulator WAPL and Sororin control the ring open and close status,^29,30^ and this structure topologically encircles chromatin. Cohesin is loaded onto chromatin in early G1 phase and established during DNA replication in S phase.^22,31^

Here we report a novel role of nuclear PD-L1 in regulation of the cohesin complex. Our findings show that PD-L1, compensating for the loss of Sororin, competes with WAPL binding to PDS5B and regulates cohesin complex status and genomic stability in cancer cells. PD-L1 deficiency suppresses tumor growth in a PD-1 independent manner. Moreover, *Pd-l1* knockout mice do not display cohesion defect, suggesting a unique role of PD-L1 in cancer cells.

## Results

### PD-L1 is required for TNBC cell proliferation and tumor growth independent of PD1

**Fig. 1:**
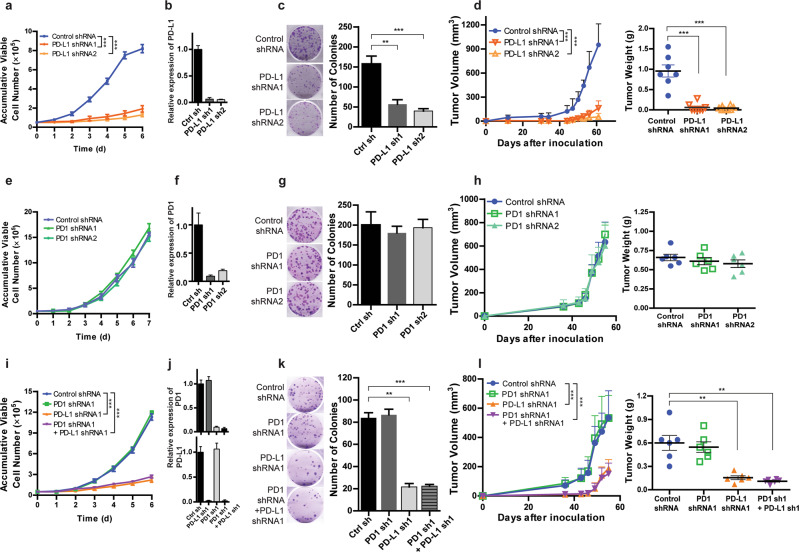
PD-L1 is required for TNBC cell proliferation and tumor growth independent of PD1. **a**–**d** PD-L1 promotes cell growth. MDA-MB-231 cells were infected with control shRNA or two different PD-L1 shRNA viruses. **a** Cell growth was monitored at indicated time points by cell counting. **b** PD-L1 knockdown efficiency was determined by qRT-PCR. **c** Colony formation assays were performed. **d** In vivo tumor growth in NSG mice was assessed and tumor weights were measured when experiments were terminated. **e**–**h** PD1 is dispensable for cell growth. **e** MDA-MB-231 cells expressing control shRNA or two different PD1 shRNAs were monitored for cell proliferation at indicated time points by cell counting. PD1 knockdown efficiency (**f**), colony formation (**g**), and tumor growth in NSG mice (**h**) were determined, respectively. **i**–**l** PD-L1-mediated cell proliferation is independent of PD1. **i** MDA-MB-231 cells expressing control shRNA, PD-L1 shRNA, PD1 shRNA, or a combination of PD-L1 shRNA and PD1 shRNA were monitored for cell growth. Knockdown efficiency (**j**) and colony formation (**k**) were independently replicated three times with similar results. **l** Tumor growth at different time points was determined and tumor weights were measured at the time when the experiments were terminated (n = 6–7). Data are presented as means ± SEM of n = 3 independent experiments. Student’s *t-*test was used for the comparisons for (**c**, **d**) (right panel), (**g**, **h**) (right panel), (**k**, **l**) (right panel), and 2-way ANOVA was used for (**a**, **d**) (left panel), (**e**, **h**) (left panel), (**i**, **l**) (left panel). **P < 0.01, ***P < 0.001.

We first suppressed PD-L1 expression in two TNBC cell lines that highly express PD-L1 to evaluate its effect on cellular phenotypes (Fig. 1a–d; Supplementary information, Fig. S1a, b). Interestingly, depletion of PD-L1 with two different shRNAs dramatically suppressed MDA-MB-231 cell proliferation and colony formation (Fig. 1a–c). To confirm this result, we also generated inducible *PD-L1* knockout cell lines. Knocking out *PD-L1* also greatly reduced colony formation in MDA-MB-231 cells (Supplementary information, Fig. S1a). A similar phenotype was observed in a second TNBC cell line, BT549 cells (Supplementary information, Fig. S1b). Based on expression of estrogen receptor (ER), progesterone receptor (PR), and human epidermal growth factor receptor 2 (HER2), breast cancer can be classified into three subtypes, including ER-positive breast cancer, HER2-positive breast cancer, and triple negative breast cancer (TNBC). Compared to TNBC cells, ER-positive or HER2-positive breast cancer cells, including MCF7, ZR-75-1, and BT474 cells, express very low levels of PD-L1. To test the effect of PD-L1 knockdown in both subtypes in addition to TNBC, we also transduced PD-L1 shRNA lentivirus into cell lines with various receptor status (Supplementary information, Fig. S1c). Interestingly, PD-L1 knockdown did not affect proliferation of these cells (Supplementary information, Fig. S1d–f), suggesting impaired cell survival is specific to cells that highly express PD-L1. PD-L1 has also been reported to be overexpressed in many different cancer types, including prostate, colon, melanoma, and ovarian cancers. To test whether our observation that PD-L1 is required for cell proliferation is generalizable to other cancers, we assessed PD-L1-mediated proliferation in cancer cell lines from different tissue origins, including lung, colon, and prostate. As expected, PD-L1 expression varied among cell lines, with several cell lines showing high PD-L1 expression (Supplementary information, Fig. S1g). Depletion of PD-L1 in these cells significantly suppressed colony formation (Supplementary information, Fig. S1h), suggesting that PD-L1 is important for proliferation in cancer cells that highly express PD-L1.

To investigate whether loss of PD-L1 affects tumor growth in vivo, we injected MDA-MB-231 cells stably expressing control shRNA or two different PD-L1 shRNAs into NSG mice. These mice lack both innate and adaptive immunity, thereby being a perfect model to exclude PD-L1’s immunosuppression effect.^32^ We found that depletion of PD-L1 in MDA-MB-231 cells greatly impeded tumor growth (Fig. 1d). These results indicate that PD-L1 contributes to TNBC cell proliferation and tumor growth.

PD1, the receptor for PD-L1, is widely expressed in many different cancers.^32^ Thus, we examined the expression of PD1 in different subtypes of breast cancer. Distinct from the PD-L1 expression profile, which is overexpressed in TNBC (when compared to hormonal receptor positive or HER2+ breast cancer subtypes), PD1 levels varied little among the cell lines across different subtypes (Supplementary information, Fig. S1c). Kleffel et al have reported that in human melanoma cells, overexpression of PD1 enhances tumor proliferation.^32^ Therefore, we explored whether PD1 might regulate cell growth and tumor progression in TNBC. We depleted PD1 in MDA-MB-231 cells using two different shRNAs, but did not observe any significant changes in cell proliferation or colony formation (Fig. 1e–g). Similar results were obtained in BT549 cells (Supplementary information, Fig. S1i). To further confirm this finding, we injected MDA-MB-231 cells stably expressing control shRNA or PD1 shRNA into immunodeficient NSG mice. Consistent with the in vitro results, depletion of PD1 did not affect tumor growth in mice (Fig. 1h). These results suggest that in contrast to PD-L1, tumor cell expression of PD1 does not drive TNBC cell growth either in vitro or in vivo.

It has been reported that tumor intrinsic PD1 can interact with PD-L1 and enhance proliferation of melanoma cells by activating the mTOR pathway.^32^ To test whether the interaction between PD-L1 and PD1 might affect TNBC cell growth, we treated MDA-MB-231 and BT549 cells with increasing concentrations of anti-PD-L1 antibody that blocked the interaction between PD-L1 and PD1, and monitored cell growth. Interestingly, no inhibition of cell proliferation was observed when the cells were treated with an anti-PD-L1 antibody (Supplementary information, Fig. S1j, k). Next, we depleted PD-L1 and PD1 either separately or together in MD-MB-231 cells (Fig. 1i–k). Consistently, knockdown of PD-L1, but not PD1, greatly suppressed cell proliferation and colony formation. Furthermore, knocking down PD1 in PD-L1 depleted MDA-MB-231 cells did not further affect either cell proliferation or colony formation (Fig. 1i–k), suggesting that the regulation of cell growth is independent of the interaction between PD-L1 and PD1 in these cells. To further confirm these results, we injected MDA-MB-231 cells stably expressing control shRNA, PD-L1 shRNA, PD1 shRNA, or PD1 shRNA plus PD-L1 shRNA into NSG mice, and monitored tumor growth. No significant difference in tumor growth was observed between the PD-L1 depleted tumor group and the PD-L1 and PD1 double knockdown group (Fig. 1l). Taken together, these results indicate that PD-L1 contributes to TNBC cell proliferation and tumor growth in a PD1 independent manner.

### PD-L1 is a cell cycle dependent protein and regulates sister chromatid cohesion in TNBC

**Fig. 2:**
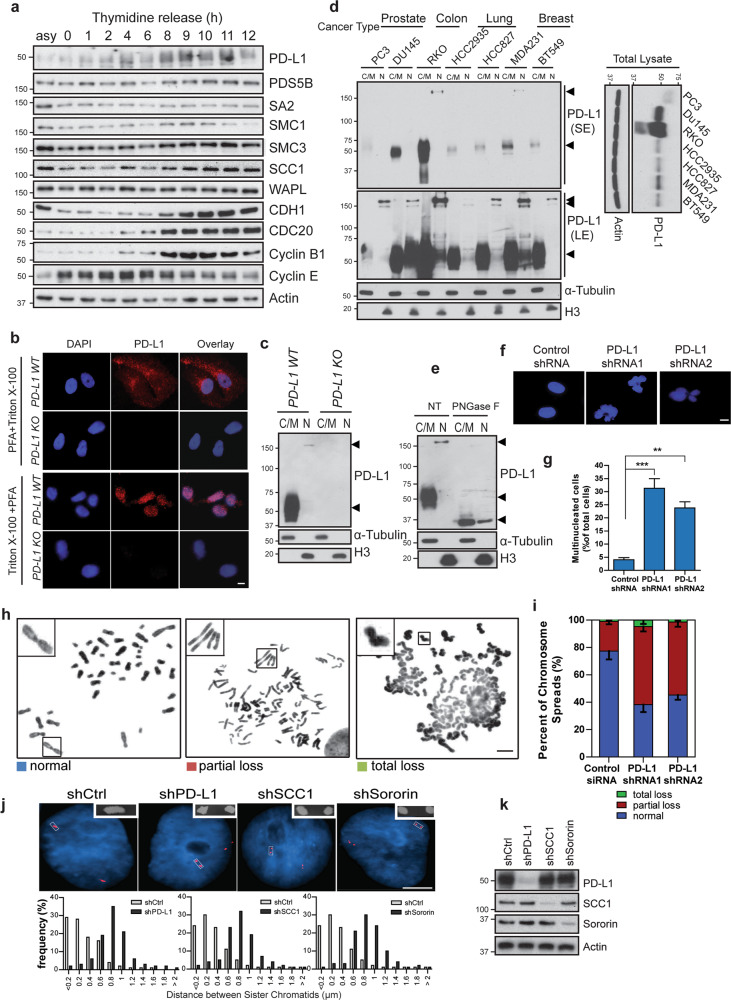
PD-L1 is a cell cycle dependent protein and regulates sister chromatid cohesion in TNBC. **a** PD-L1 level fluctuates during the cell cycle. Target proteins were detected by Western blot in MDA-MB-231 cells at indicated time points after double thymidine block. **b** Immunostaining of PD-L1 in interphase cells. Wildtype (WT) or *PD-L1* knockout (KO) MDA-MB-231 cells were permeabilized and fixed using the indicated methods and cells were then stained with anti-PD-L1 antibody. PFA + Triton X-100: cells were fixed with 3% paraformaldehyde followed by 0.5% Triton X-100. Triton X-100 + PFA: cells were treated with 0.5% Triton X-100 first to remove most cytoplasmic/membrane proteins, and followed by fixation with 3% paraformaldehyde. Nucleus was stained with DAPI. Scale bar: 10 µm. **c** Detection of PD-L1 in different fractions of control and *PD-L1* knockout MDA-MB-231 cells. Cytoplasmic/membrane (C/M) and nuclear (N) fraction. **d** Cytoplasmic/membrane (C/M) and nuclear (N) fractions of PD-L1 from different cancer cell lines. SE short exposure; LE long exposure. **e** Cytoplasmic/membrane (C/M) and nuclear (N) fractions of MDA-MB-231 cells with or without PNGase F treatment. Arrows indicate PD-L1 bands. **f**, **g** Loss of PD-L1 promotes multinucleation. **f** Representative nuclear staining by DAPI in MDA-MB-231 cells three days post-infection with control shRNA virus or PD-L1 shRNA virus. Scale bar: 10 µm. **g** Quantification of multinucleated cells was performed. Data are presented as means ± SD, and were independently replicated three times. Statistical significance was calculated using Student’s *t* test. **P < 0.01, ***P < 0.001. **h**, **i** PD-L1 is required for sister chromatid cohesion. **h** Representative metaphase spreads showing MDA-MB-231 cells with normal, partial loss and total loss of sister chromatid cohesion. Scale bar: 5 µm. **i** Quantification of different sister chromatid status in control and PD-L1 knockdown cells. Data are presented as means ± SD, independently replicated three times with similar results. **j** FISH assays were performed in MDA-MB-231 cells expressing control shRNA, PD-L1 shRNA, SCC1 shRNA or Sororin shRNA. *myb* gene probe was used and the distance between paired FISH signals was measured and quantified. Bar: 5 µm. **k** Western blots showing the knockdown efficiency for the experiments in **j**.

To better understand the role of PD-L1 in TNBC cell proliferation, we examined the protein levels of PD-L1 during different cell cycle phases. Unexpectedly, we found that PD-L1 protein accumulates in the G2/M phase and decreases in G1 (Fig. 2a). These results suggest that PD-L1 expression in TNBC is cell cycle dependent. This observation is consistent with a recent report by Zhang et al which showed similar cell cycle dependent changes in PD-L1.^33^ Detection of PD-L1 signals in the nucleus by immunofluorescence labeling after permeabilization with Triton-X-100 suggested that PD-L1 protein associates with DNA (Fig. 2b). To further confirm the nuclear localization of PD-L1, we performed fractionation assays and detected PD-L1 in the nuclear fraction (Fig. 2c). Surprisingly, we found that nuclear PD-L1 was super-shifted (over 150 kDa) compared to the cytosolic/membrane fraction by Western blot, and this band was absent in *PD-L1* knockout cells (Fig. 2c). The same super-shift bands for nuclear PD-L1 were observed in multiple cancer cells of different origins (Fig. 2d). Treatment of the therapeutic PD-L1 antibody, which blocks PD1/PD-L1 interaction, did not change PD-L1 protein level in both cytosolic/membrane fraction or nuclear fraction (Supplementary information, Fig. S1l). This might explain the lack of inhibition of cell proliferation when the cells were treated with PD-L1 blocking antibody (Supplementary information, Fig. S1j, k).

It has previously been reported that PD-L1 is N-glycosylated and this product has a much higher molecular weight at 50–70 kD detected by Western blot.^34^ PNGase F (peptide-N-glycosidase F) treatment can release the N-glycans attached to asparagine residues on PD-L1 and shift the band down to a lower molecular weight, ~35 kD. When we treated both nuclear and cytosolic/membrane fractions with PNGase F, we found that PD-L1 protein in both fractions shifted down to ~35 kD (Fig. 2e), suggesting that nuclear PD-L1 is a full length protein and is more highly glycosylated compared to the cytosolic/membrane form. Glycosylation of substrates in the Golgi is reported to assist nuclear localization of proteins.^35^ Therefore, these results suggest that PD-L1 might be directly transported into the nucleus after being synthesized and glycosylated in the ER and Golgi apparatus.

Interestingly, we noticed a significant increase in the number of multinucleated cells after PD-L1 knockdown (Fig. 2f, g), but not after PD1 depletion (Supplementary information, Fig. S2a). Double knockdown of both PD1 and PD-L1 in MDA-MB-231 cells did not further increase the proportion of multinucleated cells (Supplementary information, Fig. S2b). Analysis of the chromosome structure by chromosome spreads assay showed that the kinetochore region of sister chromatids in control cells were easily detectable. However, in the PD-L1 knockdown cells, approximately 50% of sister chromatid arms were partially separated and total loss of cohesion between the two sister chromatids was observed in a fraction of cells (Fig. 2h, i). To analyze sister chromatid cohesion, FISH assay was performed and the distance between the paired *myb* gene was measured as described previously.^26^ Compared to control cells, the average inter-chromatid distance in PD-L1 depleted cells was much wider, a phenotype similar to that observed after depletion of SCC1, one of the components of the cohesin complex (Fig. 2j, k). Consistent results were detected in three additional *PD-L1* knockout cells from different tumor types (Supplementary information, Fig. S3a–c). Collectively, these results suggest that PD-L1 may be involved in regulation of the cohesin complex in cancer cells.

### PD-L1 interacts with cohesin complex

**Fig. 3:**
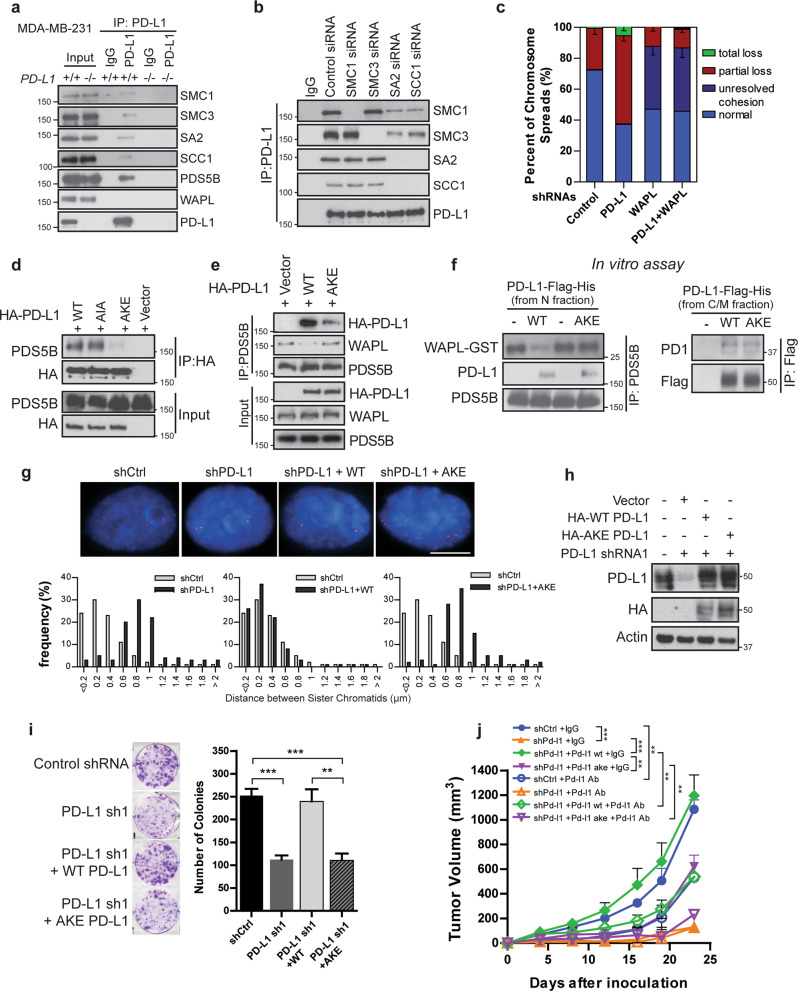
Nuclear PD-L1 interacts with cohesion complex. **a**, **b** PD-L1 interacts with cohesin complex. **a** Doxycycline-inducible *PD-L1* widetype (WT) (+/+) and knockout (KO) (−/−) MDA-MB-231 cells were harvested and fractionated. The nuclear fraction was incubated with monoclonal anti-PD-L1 antibody or corresponding IgG. The immunoprecipitates were blotted with indicated antibodies. **b** Cells were blocked with thymidine and transfected with indicated siRNAs. After releasing from the second round of thymidine block, cells were released into S phase in the presence of MG132 for 3 h. Cells were harvested and nuclear fractions were processed for immunoprecipitation. **c** PD-L1 is involved in the regulation of cohesion in the presence of WAPL. MDA-MB-231 cells expressing control shRNA, PD-L1 shRNA, WAPL shRNA, or a combination of PD-L1 and WAPL shRNAs were fixed and dropped on coverslips. Percentages of cells with different sister chromatid cohesion status were quantified. Data are presented as means ± SEM, and were independently replicated three times. **d**–**f** PD-L1 YSR-like motif (YKR) is essential for PD-L1 binding with PDS5B in vivo, and competes with WAPL for binding to PDS5B in vitro. PD-L1 wild-type (WT), PD-L1 AIA mutant (mutating both FGF-like motifs to AIA), and PD-L1 AKE mutant (mutating YKR to AKE) plasmids were transfected into cells and nuclear fractions were isolated and incubated with (**d**) HA-conjugated sepharose beads of (**e**) protein A beads conjugated with anti-PDS5B antibody. The immunoprecipitates were blotted with indicated antibodies. **f** Left panel: in vitro competition of binding affinity of PDS5B (purified from insect cells) and purified WAPL (1–30)-GST and PD-L1 (purified from nuclear fraction of MDA-MB-231 cells). Right panel: in vitro interactions between PD1 and wildtype (WT) or AKE mutant PD-L1 purified from cytoplasmic/membrane fraction (C/M) of MDA-MB-231 cells. **g**–**j** PD-L1 YSR-like motif is essential for sister chromatid cohesion and cell proliferation. *myb* gene distance between paired FISH signals (**g**), and colony formation (**i**) were measured in MDA-MB-231 cells infected with control shRNA, PD-L1 shRNA, PD-L1 shRNA plus WT shRNA resistant PD-L1 overexpression construct, or PD-L1 shRNA plus shRNA resistant AKE mutant PD-L1 overexpression construct. Scale bar: 10 µm. Data are presented as means ± SEM, and were independently replicated three times with similar results. Statistical significance was calculated using Student’s *t-*test. **P < 0.01, ***P < 0.001. **h** Protein levels in cells used for assays in (**g** and **i**) were detected by Western blot with indicated antibodies. **j** B16F10 cells expressing control shRNA (shCtrl), mouse Pd-l1 shRNA (shPd-l1), Pd-l1 shRNA plus shRNA resistant mouse wildtype Pd-l1 (shPd-l1 + Pd-l1 wt), or mouse Pd-l1 shRNA plus shRNA resistant ake mutant Pd-l1 (shPd-l1 + Pd-l1 ake) were injected into C57BL/6 mice. Mice were treated with 5 mg/kg mouse Pd-l1 blocking antibody or corresponding IgG. The tumor growth was monitored by measuring with a caliper every 3–4 days. Data are presented as means ± SEM (n = 5). Two-way ANOVA was used for the comparisons. **P < 0.01, ***P < 0.001.

The cohesin complex contains four core subunits, SMC1, SMC3, SA2 and SCC1 (also known as RAD21) and forms a ring-shaped structure that traps DNA inside.^36^ The cohesin complex secures correct sister-chromatid segregation during mitosis and meiosis and maintains genome integrity.^37–39^ WAPL drives cohesin release from chromatin by opening a distinct DNA exit gate at the interface connecting the SMC3 and SCC1 subunits.^40,41^ On the other hand, Sororin antagonizes WAPL and maintains sister chromatid cohesion.^26^ Based on the above observations, we were interested in testing whether PD-L1 might be directly connected to the cohesin complex. Specifically, we carried out immunoprecipitation assays and examined interactions between nuclear PD-L1 and the cohesin complex in the nuclear fraction. We detected all four subunits of the cohesin complex, SMC1, SMC3, SA2, and SCC1, in the PD-L1 immunoprecipitates, but not in IgG or *PD-L1* knockout immunoprecipitates (Fig. 3a). Similar interactions were also detected in additional cancer cell lines expressing high levels of PD-L1 (Supplementary information, Fig. S4a). To determine which subunit of the cohesin complex directly interacts with nuclear PD-L1, we depleted each individual subunit by using siRNA and pulled down nuclear PD-L1 to detect interacting proteins. When we knocked down SMC1 or SMC3 in cells with specific siRNAs, the interaction of PD-L1 with the other subunits did not change. However, when we depleted SA2 or SCC1, PD-L1 interaction with the cohesin complex was disrupted (Fig. 3b and Supplementary information, Fig. S4b), indicating that PD-L1 interacts with the cohesin complex through the SA2 and SCC1 subunits. Importantly, knocking down individual components of the cohesin complex did not affect the expression of the remainder of the complex (Supplementary information, Fig. S4b)

WAPL is the protein responsible for opening the hinge of the cohesin complex and maintaining the dynamic binding state. Overexpression of WAPL causes cohesion defects similar to what we observed in PD-L1 depleted cells.^25,42^ To determine the relationship between WAPL and PD-L1, we knocked down WAPL and PD-L1 both separately and simultaneously, and examined the chromosome cohesion. As expected, knockdown of WAPL led to tightly connected sister chromatids. Depletion of WAPL and PD-L1 simultaneously caused a phenotype similar to WAPL depletion alone (Fig. 3c), suggesting that PD-L1 regulates cohesion in the presence of WAPL. However, we did not detect an interaction between PD-L1 and WAPL. Surprisingly, PDS5B, a competitive binding partner for both WAPL and Sororin, was detected in the PD-L1 immunoprecipitates (Fig. 3a). Based on these observations, we hypothesized that PD-L1 might compete with WAPL for its binding to PDS5B. Two PDS5B binding motifs, the FGF motif and the YSR motif, have been previously identified.^24,26,43^ We examined the protein sequence of PD-L1 and found two FGFlike motifs and one YSR-like motif (Supplementary information, Fig. S4c, d). Unlike the FGF-like motif, the YSR-like motif was conserved across species. While mutation of the FGF-like motif did not affect the binding between PD-L1 and PDS5B, mutating the highly conserved YKR residues within the YSR-like motif to AKE (amino acids YKR mutated into AKE) greatly decreased the binding of PD-L1 to PDS5B (Fig. 3d). We also observed that wild-type PD-L1 expression decreased the interaction between PDS5B and WAPL, while the PD-L1 AKE mutant did not (Fig. 3e). This suggests a competition between PD-L1 and WAPL in binding to PDS5B. In vitro competition assays showed that purified wildtype PD-L1, but not the AKE mutant from nuclear fraction, was able to compete with WAPL for its binding to PDS5B (Fig. 3f). Meanwhile, both the wild-type PD-L1 and purified AKE mutant from the cytosolic/membrane fraction interacted with PD1 (Fig. 3f, right panel), further supporting our finding that the nuclear function of PD-L1 is independent of its function as the ligand for PD1 (Fig. 1e–l). To further evaluate this competition functionally, we reintroduced WT PD-L1 or the AKE mutant into PD-L1 knockdown cells, and found that expression of WT PD-L1, but not the mutant PD-L1, restored sister chromatids cohesion, cell proliferation, and colony formation **(**Fig. 3g–i). Similar data were obtained using the *PD-L1* knockout MDA-MB-231 cells (Supplementary information, Fig. S4e), suggesting that PD-L1 competes with WAPL binding to PDS5B and enhances sister chromatid cohesion. To further test whether the AKE mutation affects tumor growth and the immune suppression function of PD-L1, we injected mouse B16F10 cells expressing control shRNA, mouse Pd-l1 shRNA, mouse Pd-l1 shRNA plus shRNA resistant wildtype Pd-l1, or mouse Pd-l1 shRNA plus shRNA resistant ake Pd-l1 into immunocompetent C57BL/6 mice. These mice were treated with mouse Pd-l1 blocking antibody or control IgG antibody. Consistent with the results obtained in MDA-MB-231 xenografts (Fig. 1d), loss of Pd-l1 suppressed tumor growth (Fig. 3j). Restoration of wildtype Pd-l1 rescued tumor growth. Consistent with the colony formation results obtained in human cancer cells, reintroduction of the ake mutant impaired tumor growth when compared to the wildtype Pd-l1 expressing xenografts (Fig. 3j). Furthermore, mouse Pd-l1 blocking antibody suppressed the growth of tumor expressing control shRNA, but had no effect on Pd-l1 depleted tumors (Fig. 3j). Consistently, this blocking antibody inhibited tumor growth in Pd-l1 depleted xenografts restored with the wildtype Pd-l1 or the ake mutant, regardless of the fact that xenograft tumors expressing wildtype Pd-l1 grew much faster than tumors expressing the ake mutant (Fig. 3j and Supplementary information, Fig. S5a, b). These results suggest that the PD-L1 AKE mutant impairs PD-L1 function in the nucleus while still maintaining its immunosuppressive effect.

### PD-L1 compensates for the loss of Sororin and regulates sister chromatid cohesion

The function of PD-L1 is reminiscent of that of Sororin—to compete with WAPL for binding to PDS5B and to stabilize cohesion on DNA.^28^ To further determine the biological implication of this observation, we first examined the expression of Sororin in breast cancer cell lines of different subtypes. Surprisingly, Sororin was more highly expressed in Luminal A, Luminal B, and HER2+ cells and less well expressed in TNBC cells, a pattern opposite to that of PD-L1 (Supplementary information, Fig. S1c). We also determined PD-L1 and Sororin expression in human breast cancer tissues by tissue microarray (TMA), and found that tumor PD-L1 expression was negatively correlated with Sororin expression in TNBCs (Supplementary information, Fig. S6a–c). Based on these observations, we hypothesized that PD-L1 may regulate chromatid cohesion and compensate for the defect caused by the loss of Sororin.

**Fig. 4:**
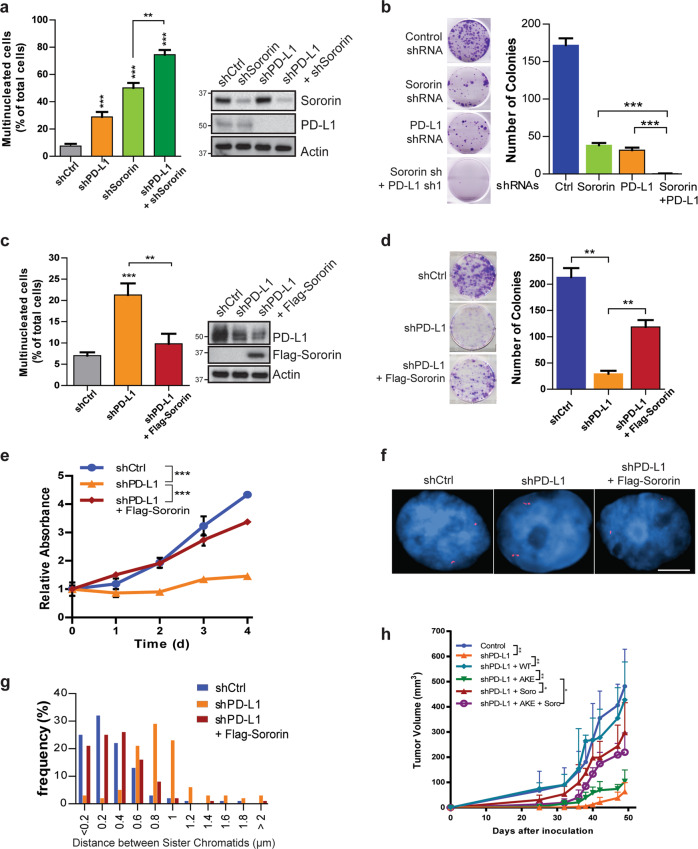
PD-L1 compensates for the loss of Sororin and regulates sister chromatid cohesion. **a**, **b** Effect of double knockdown of PD-L1 and Sororin on various cellular phenotypes in MDA-MB-231 cells. Quantification of multinucleated cells (**a**) and colony formation (**b**) in cells expressing control shRNA, PD-L1 shRNA, Sororin shRNA, or combined PD-L1 shRNA and Sororin shRNA, respectively. **c**–**h** Overexpression of Sororin reversed various cellular phenotypes caused by depletion of PD-L1 in MDA-MB-231 cells. Quantification of multinucleated cells (**c**), colony formation (**d**), cell proliferation (**e**), representative images of *myb* gene distance between paired FISH signals (**f**), and quantification of *myb* gene distance between paired FISH signals (**g**), tumor growth in NSG mice xenografted with cells expressing control shRNA, PD-L1 shRNA, PD-L1 shRNA plus WT or AKE PD-L1, PD-L1 shRNA plus Sororin, or PD-L1 shRNA + Sororin + AKE PD-L1 expressing plasmid (**h**). For cell experiments, data are presented as means ± SEM, and were independently replicated three times with similar results. Statistical significance was calculated using 2-way ANOVA in **e**. Statistical significance was calculated using Student’s *t-*test in **a**–**d**. For experiments related to the xenograft mice models, tumor growth data are presented as means ± SD (n = 5) and 2-way ANOVA was used for the comparisons in **h**. *P < 0.05, **P < 0.01, ***P < 0.001.

To test this hypothesis, we knocked down PD-L1 and Sororin separately or together and examined the number of multinucleated cells. We found that depletion of Sororin or PD-L1 individually greatly induced the formation of multinucleated cells. Depletion of both proteins simultaneously resulted in even more multinucleated cells when compared to individual knockdowns (Fig. 4a). We also performed colony formation assay, and found that knockdown of both PD-L1 and Sororin showed a greater suppression of colony formation when compared with single gene knockdown (Fig. 4b). Taken together, these results suggest that PD-L1 and Sororin may function independently.

To further confirm the independent roles of PD-L1 and Sororin, we overexpressed Sororin in PD-L1 knockdown MDA-MB-231 cells, and found that introducing Sororin decreases the number of multinucleated cells caused by the loss of PD-L1 (Fig. 4c). We further observed restoration of cell proliferation, colony formation, as well as sister chromatid cohesion upon overexpression of Sororin in PD-L1 depleted MDA-MB-231 cells (Fig. 4d–g). We also injected NSG mice with control cells, PD-L1 knockdown cells, and PD-L1 knockdown cells ectopically expressing Sororin. Consistently, loss of PD-L1 significantly impaired tumor growth, but overexpression of Sororin alleviated this impairment (Fig. 4h), suggesting that PD-L1 compensates for the function of Sororin in TNBC cells, which have low Sororin expression. Finally, we also ectopically expressed PD-L1 in Sororin depleted BT474 cells. As expected, we found that the loss of Sororin greatly suppressed colony formation, while the introduction of PD-L1 rescued this growth defect (Supplementary information, Fig. S7).

### Normal sister chromatid cohesion in *Pd-l1* knockout mice

**Fig. 5:**
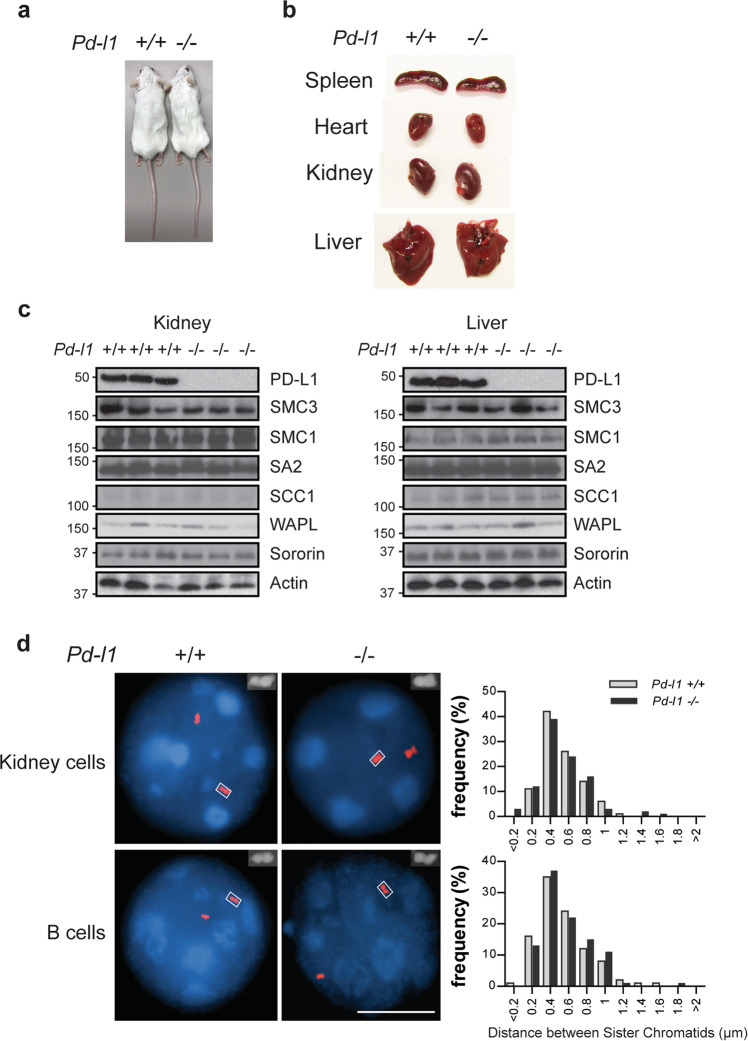
*Pd-l1* knockout mice display normal phenotypes including sister chromatid cohesion. **a** Image of *Pd-l1* WT (+/+) and KO (−/−) mice. **b** Comparison of organ sizes between the *Pd-l1* WT (+/+) and KO (−/−) mice. **c** Protein expression of cohesin subunits in kidney or liver tissues from the *Pd-l1* WT (+/+) and KO (−/−) mice. **d** Representative images of FISH assays to determine sister chromatid cohesion in primary kidney cells and B cells isolated from the *Pd-l1* WT (+/+) and KO (−/−) mice. Scale bar: 10 µm.

To better understand nuclear PD-L1 function in normal cell homeostasis, we utilized *Pd-l1* knockout mice.^44^ We did not observe a significant growth defect in *Pd-l1* knockout mice (Fig. 5a, b). We also did not find any changes in protein levels of cohesin complex subunits in *Pd-l1* knockout mice when compared to wildtype mice (Fig. 5c). Moreover, FISH assay using primary mouse cells did not reveal significant changes in the distance between sister chromatids (Fig. 5d). These results suggest that PD-L1 may not be involved in cohesion regulation in normal cells. This could be due to the fact that Sororin is maintained at normal protein levels under normal conditions.

## Discussion

Previous studies have reported that tumor PD-L1 interacts with its receptor PD1, suppresses the development of the T cell response and evades anti-tumor immunity.^6,7^ Moreover, increasing evidences demonstrate that both PD-L1 and PD1 also play intrinsic roles in cancer cell signaling. Intrinsic PD1 in melanoma cells regulates mTOR signaling and promotes tumor growth.^32^ Intrinsic PD-L1 is also implicated to be involved in different biological processes, such as autophagy and glucose metabolism.^14–16^ Here we found that PD-L1 is overexpressed in multiple different cancers including triple negative breast cancer, which portends a poor prognosis (Supplementary information, Fig. S1c). In addition, we also detected nuclear localization of PD-L1 protein (Fig. 2b, c). In spite of extensive studies on its function as a cell membrane protein, little is known about PD-L1 as a nuclear protein. The membrane protein detected in the nucleus is not an exception for PD-L1. There is a substantial body of evidence documenting other membrane proteins e.g. epidermal growth factor receptor (EGFR) family members to be localized in the nucleus.^45,46^ The translocation of EGFR family members involves the recognition of the nuclear localization signal (NLS) by the nuclear transporter importin proteins, thus directing the proteins transport through the nuclear pore complex to the nucleus.^47–49^ However, unlike EGFR, PD-L1 does not contain a nuclear localization sequence. Post-translation modifications in the ER and trans-Golgi network, including glycosylation, have been implicated in trafficking of various proteins to the plasma membrane or cell nucleus.^50,51^ Previous studies and the current study have shown that PD-L1 is exclusively N-glycosylated (Fig. 2e).^34^ Compared to the plasma membrane PD-L1 (~50 kD shown on immunoblot), the nuclear PD-L1 (>150 kD on immunoblot) may involve more complex glycan forms (Fig. 2c–e), suggesting that nuclear PD-L1 is less likely directly internalized from plasma membrane. It is possible that PD-L1 is heavily glycosylated after translation in the ER and Golgi apparatus, and that this heavily glycosylated form of PD-L1 is recognized by glycosylation binding proteins and transported into the nucleus. The exact mechanisms involved in the process of nuclear PD-L1 localization requires further investigation.

Here we report that nuclear PD-L1 interacts with cohesin complex and regulates sister chromatid cohesion (Figs. 2h–i and 3a–g). Mechanistically, nuclear PD-L1 competes with WAPL for its binding to PDS5B through its YSR like motif and stabilizes the cohesin ring (Fig. 3d–f). This process is reminiscent of Sororin which also contains a YSR motif and competes with WAPL for binding to PDS5B.^43^ Another protein, Haispin also contains a similar motif and protects centromeric cohesion by antagonizing WAPL.^52^ In our study, we have identified that nuclear PD-L1 functions similarly to Sororin to protect sister chromatid cohesion and prevent cell death from lack of Sororin. Loss of PD-L1 greatly impairs sister chromatid cohesion (Figs. 2h–j and 3a) and suppresses tumor growth (Figs. 1a–d and 3j). These phenotypes are not limited to triple negative breast cancer and are also detected in other cancer cells from different tissue origins with high PD-L1 expression (Fig. 2d; Supplementary information, Figs. S1h, S3, and S4a). Data from *Pd-l1* knockout mouse model show that PD-L1 is not essential for the maintenance of normal tissue homeostasis (Fig. 4a, b). Disruption of PD-L1 does not affect chromatid cohesion function due to normal Sororin protein level (Supplementary information, Fig. S4c, d).

YSR like motif is localized in the PD-L1 extracellular domain. This domain is essential for the interaction between PD-L1 and its receptor, PD1 and immune checkpoint signaling.^53^ However, we found that mutation of YSR like motif did not affect membrane PD-L1 interaction with PD1 (Fig. 3f), and the mutant PD-L1 still maintained PD-L1 immunosuppression effect in vivo (Fig. 3j; Supplementary information, Fig. S5a, b).

**Fig. 6:**
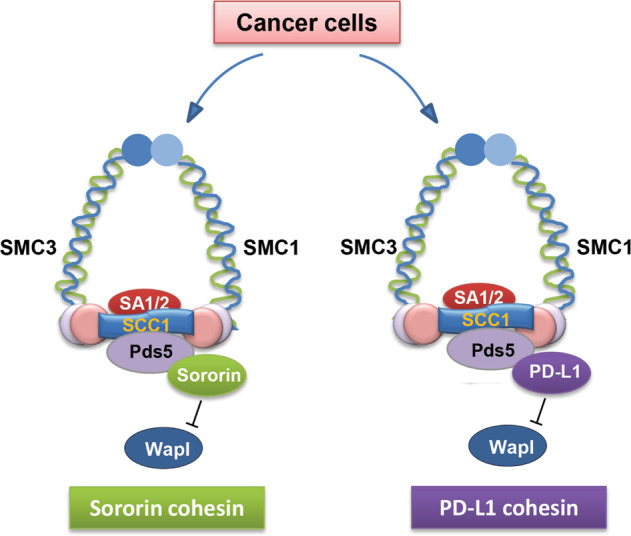
Working model. Sororin suppresses WAPL and maintains sister chromatid cohesion in Sororin cohesin cells. In contrast, in PD-L1 overexpressed cells, PD-L1 compensates for the loss of Sororin and competes with WAPL binding to PDS5B, thereby regulating sister chromatid cohesion.

Based on these findings, we propose a working model that in cancer cells with normal Sororin expression, Sororin antagonizes WAPL and controls proper sister chromatid cohesion (Fig. 6). In contrast, in TNBC or other cancers with low Sororin expression, PD-L1 overexpression is one of the mechanisms that compensates for the loss of Sororin. PD-L1 antagonizes WAPL binding to PDS5B in the cohesin complex, leading to proper chromosome segregation and cell proliferation. This observation can be extended to other cancers such as lung, colon and prostate cancers, in which the PD-L1-cohesion axis also exists. This might be one of the adaptive mechanisms for Sororin-low cancers to maintain cell proliferation, and at the same time, evade immune surveillance.

Taken together, we have reported an important and novel function of nuclear PD-L1 in the regulation of chromatid cohesion. Nuclear PD-L1 is important for establishment of the cohesin complex, controlling proper chromosome segregation and maintaining genomic integrity. Targeting nuclear PD-L1, which does not affect normal cells, may clinically benefit cancer patients with high tumor PD-L1 levels.

## Methods

### Cell culture, synchronization and transfection

The cell lines MDA-MB-231, Hs 578T, MCF7, ZR75-1, BT474, BT-549, RKO, HCC837, B16F10 and DU145 were cultured in DMEM or RPMI1640 containing 10% FBS. Breast cancer cells expressing shRNAs were generated by infecting the cells with individual shRNA virus followed by selecting resistant clones in growth medium supplemented with puromycin. Rescue experiments were conducted by transducing both shRNA lentivirus and shRNA resistant CDS expressing lentivirus into cells and selecting with puromycin for one day. For synchronization, MD-MB-231 cells were cultured in the presence of 2 mM thymidine (Sigma) for 24 h, washed twice with pre-warmed PBS and released in a fresh medium for 8 h. Thymidine was added again to a final concentration of 2 mM to block cells at G1/S. After another 16 h incubation, cells were released. For Scc1 and Sororin knockdown, 30 nM (single RNAi experiments) or 60 nM (double RNAi experiments) siRNAs were pre-mixed with Lipofectamine RNAiMax (Life Technologies) according to the manufacturer’s instructions and were added either before the first thymidine arrest (Scc1) or during the first release (Sororin), as described previously.^26^

### Plasmids and reagents

PD-L1 sgRNA plasmids and inducible Cas9 expression plasmids were purchased from Dharmacon. Mouse Wildtype and AKE mutant PD-L1 coding sequences were inserted into the mouse pGIPZ–PD-L1 shRNA plasmid to replace the GFP coding sequencing. Flag-PD-L1 plasmids were purchased from Origene. Point mutations in PD-L1 were generated using the Quick-change site-directed mutagenesis kit (Stratagene) and sequences were verified by Sanger sequencing. Expression plasmids for various proteins were cloned into a pCMV plasmid (Clontech Laboratories, Inc.) with a hemagglutinin (HA) tag. Sororin plasmid was purchased from Origene.

Anti-SMC1 antibody (4802S), anti-SMC3 antibody (5696S), anti-SCC1 antibody (4321S), anti-STAG2 antibody (5882S), anti-WAPL antibody (77428S) and anti-PD-L1 antibody (13684S, for Western blot) were purchased from Cell Signaling. Anti-PD-L1 antibody (29E.2A3) for immunoprecipitation and human cell PD-L1 blocking antibody were purchased from Biolegend. Anti-Sororin antibody (ab192237), anti-SMC1 antibody (ab140493), anti-Na/K ATPase antibody (Ab76020), anti-CENPA antibody (ab13939) and anti-PD1 antibody (ab52587) were purchased from Abcam. Anti-CDH1 antibody (sc-56312), anti-CDC20 antibody (sc-136024), anti-cyclin E antibody (sc-271348), anti-PCNA antibody (sc-56) and anti-cyclin B1 antibody (sc-70898) were purchased from Santa Cruz. Anti-β-actin antibody was purchased from Sigma. Anti-PDS5B antibody was purchased from Bethyl laboratories; anti-mouse PD-L1 blocking antibody (10F.9G2) and corresponding IgG were purchased from Bioxcell.

### Generation of inducible *PD-L1* knockout cells

MDA-MB-231 cells were infected with inducible Cas9 plasmid and selected with 5 µg/mL blasticidin for two weeks in Tet-free medium. The cells were then infected with *PD-L1* sgRNA lentivirus and selected with both blasticidin and puromycin. Single colonies were picked up and screened for *PD-L1* knockout with doxycycline induction.

### Animals

All animal work was approved by the Mayo Clinic Institutional Animal Care and Use Committee (IACUC). 6–8 week-old female immune-deficient NSG (NOD.Cg-Prkdcscid Il2rgtm1Wjl/SzJ) mice and 6–8 week-old female C57BL/6 mice were ordered from Jackson Laboratories. *Pd-l1* knockout mice were maintained in a 12 h light/dark cycle, and fed ad libitum normal food.

### Xenograft models

MDA-MB-231 cells stably expressing control shRNA, shRNAs targeting PD-L1 or PD1 genes, or cells overexpressing PD-L1 or indicated genes were mixed in PBS 1:1 with matrigel (BD Bioscience). Cells (1 × 10^6^ cells/mouse) were then injected subcutaneously into 6–8 week-old female immune-deficient NSG (NOD.Cg-Prkdcscid Il2rgtm1Wjl/SzJ) mice. B16F10 stably expressing control shRNA, mouse Pd-l1 shRNA, mouse Pd-l1 shRNA plus shRNA resistant wildtype Pd-l1, or mouse Pd-l1 shRNA plus shRNA resistant ake mutant Pd-l1 were injected subcutaneously into C57BL/6 mice and were treated with 5 mg/kg mouse Pd-l1 blocking antibody twice a week. Tumors were measured with a caliper on the indicated days. Tumor volumes were calculated as described previously.^54,55^ Mice were sacrificed and tumors were dissected when they met the criteria set by IACUC. Data were analyzed using ANOVA or Student’s *t*-test.

### Clonogenic assay

For clonogenic assays, breast cancer cells were seeded in triplicate in 6-well plates at 500 or 1000 cells per well. Ten to 15 days later, cells were fixed with 100% methanol for 5 min, followed by 0.1% (w/v) Giemsa. Colonies with more than 50 clones were counted on a GelCount (Oxford Optronix).

### Fluorescence in situ hybridization (FISH)

Prior to processing, slides were aged on a 90 °C ThermoBrite™ for 5 to 10 min. Home-brewed human *MYB* DNA clones (521H20, RP1-32B1, RP3-388E23 and RP1-71N10) or mouse clones, 4qE2 (RP23-182D5, RP23-149J22, RP24-256N4, RP24-231I13) probe cocktail were used for FISH to detect sister chromatids’ signals in human or mouse cells. Probes labeled with SpectrumOrange dUTP (Abbott Molecular/Vysis Products) were combined into one probe, respectively. The probe was then applied onto individual slides, hybridized, and washed according to the Interphase FISH protocol. Specifically, 5–10 μL of the DNA probe working solution was applied to selected hybridization areas on the pre-treated slides, and then coverslipped and denatured using ThermoBrite (Abbott Molecular) at 75 °C for 5 min, followed by hybridization for 20 h in a 37 °C humidified oven.

Post-hybridization wash was performed first with pre-warmed 0.4 × SSC (saline-sodium citrate) for 2 min at 74 °C, followed by 0.1% NP-40/2 × SSC solution in a dark environment at room temperature for 1 min. The slides were stained with DAPI, 4′-6,-diamidino-2-phenylindole (Vector Laboratories, Burlingame, CA) and coverslipped. Visualization and acquisition of images of the FISH signals were performed by Cytovision (Leica Biosystems, Buffalo Grove, Illinois). FISH signals representing distance between sister chromatids were measured in Image J. On average, more than 200 cells were measured and counted for each experiment.

### Cell fractionation assay

For chromatin isolation, cells were harvested and lysed with 10 volumes of NETN buffer with low salt (20 mM Tris-HCl, pH 8.0, 10 mM NaCl, 1.5 mM MgCl_2_, 1 mM EDTA, 0.5% Nonidet P-40, 20 mM NaF, 1 mM Na_3_VO_4_, 1 μg/mL aprotinin, and 1 μg/mL pepstatin). The chromatin-enriched pellet was washed with PBS three times and resuspended in 0.2 M HCl for 30 min on ice. The soluble extract was neutralized with 1 M NaOH for further analysis. Cytoplasmic/Membrane and nuclear fractions were separated using the Paris Kit (Ambion). Membrane, cytoplasmic and nuclear fractions were isolated by Plasma Membrane Protein Extraction Kit (Abcam).

### Chromosome spreading

Cells were cultured with colcemid solution overnight (1 µg/mL). Cells were harvested by mitotic shake off and mixed with hypotonic solution (0.8% sodium citrate hypotonic: 0.075 M potassium chloride hypotonic, pH 6.0) at room temperature for 20–25 min. The cell mixtures were then washed three times with freshly made Carnoy’s solution (methanol to glacial acetic acid 3:1). Cells in Carnoy’s solution were then dropped onto glass slides and dried at 37 °C. Slides were then stained with pH 6.8, 5% Giemsa (Merck) for 10 min and washed carefully with tap water.

### RNA interference

PD-L1 shRNA sh1 (target sequence GACCTATATGTGGTAGAGTAT) and sh2 (target sequence CGAATTACTGTGAAAGTCAAT), and PD1 shRNA sh1 (target sequence GCCTAGAGAAGTTTCAGGGAA) and sh2 (target sequence (CGTGCTAAACTGGTACCGCAT), were purchased from Sigma. Lentivirus was produced according to manufacturer’s protocol. siRNAs targeting SMC1, SMC3, SA2 and SCC1 were purchased from Dharmacon.

### Western and Immunoprecipitation (IP) assay

Cells were lysed with 0.5% NP40, 150 mM NaCl, 50 mM Tris, and 1 mM EDTA (NETN) buffer containing 20 mM Tris-HCl (pH 8.0), 100 mM NaCl, 1 mM EDTA, 0.5% Nonidet P-40 with 50 mM b-glycerophosphate, 10 mM NaF, and 1 mg/mL each of pepstatin A and aprotinin and sonicated. After centrifugation, the supernatant was removed and incubated with indicated antibodies and protein A or protein G Sepharose beads (Amersham Biosciences) for 2 h or overnight at 4 °C.^56^ The samples were then analyzed by SDS-PAGE following three rinses with NETN buffer. Western blots were performed following standard procedures.

### Immunofluorescent staining

Cells were cultured on coverslips, and washed twice with pre-warmed PBS. For regular PD-L1 staining, cells were fixed with 3% paraformaldehyde for 10 min followed by 0.5% triton (PFA + Triton X-100) for 5 min. To better detect the nuclear PD-L1, cells were first permeabilized with 0.5% Triton X-100 for 5 min at room temperature to remove major amount of PD-L1 protein in the cytoplasm and cell membrane, and then fixed with 3% paraformaldehyde for 10 min. Cells were blocked with 5% goat serum and then incubated with PD-L1 antibody for 1 h at room temperature. After washing with PBS three times, Alexa-568-conjugated anti-rabbit secondary antibody or Alexa-488-conjugated anti-mouse secondary antibody (Jackson ImmunoResearch) was added. This was incubated for 30 min at room temperature. The cells were then counterstained with DAPI to visualize nuclear DNA. Coverslips were mounted onto glass slides with mounting solution and visualized by fluorescence microscope. The nuclear PD-L1 intensity was quantified using ImageJ software.

### Cell proliferation assay

Cells expressing different shRNAs were seeded in 6-well cell culture plates in triplicate at a density of 5000 cells per well in 2 mL medium supplemented with 10% FBS. The medium was changed every day. The cell number at the indicated time points was determined by counting using a hemocytometer.

### Statistical analysis

Data in bar or line graphs are presented as means ± SD or means ± SEM of at least three independent experiments. Comparisons were carried out with ANOVA or unpaired Student’s *t-*test (*P < 0.05, **P < 0.01, ***P < 0.001).

## Supplementary information

Supplementary FigS1

Supplementary FigS2

Supplementary FigS3

Supplementary FigS4

Supplementary FigS5

Supplementary FigS6

Supplementary FigS7
