# Supplementary material for: Regulation of sister chromatid cohesion by nuclear PD-L1
Source: Cell Res. 2020 Apr 29;30(7):590–601. doi: 10.1038/s41422-020-0315-8 (PMC7343880; doi:10.1038/s41422-020-0315-8)
Supplement: Supplementary file 1 — Supplementary FigS1 [file 41422_2020_315_MOESM1_ESM.pdf]

Supplementary Information, Fig. S1.

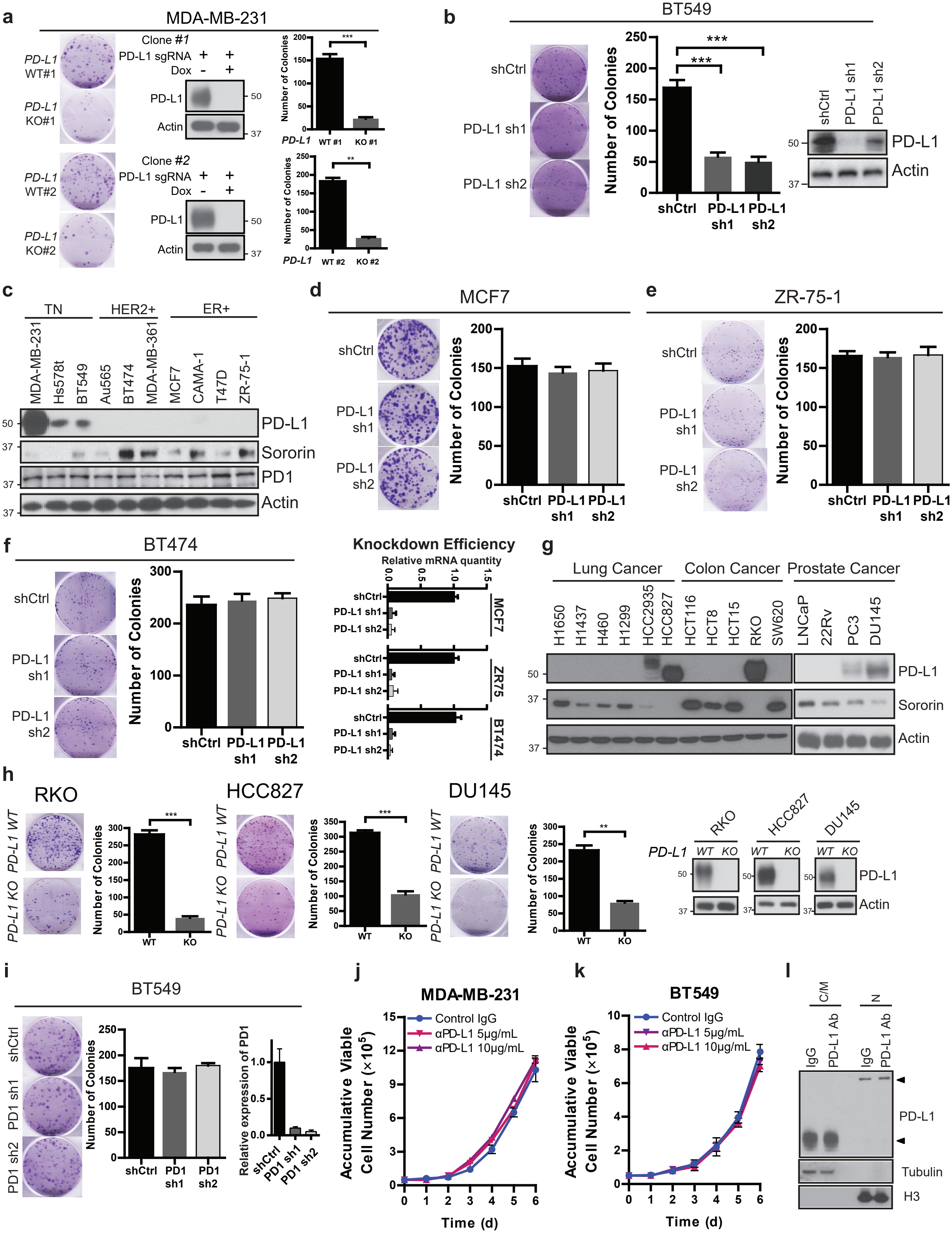

Supplementary Information, Fig. S1. PD-L1 is required for TNBC cell proliferation and tumor growth, independent of PD1.

(**a**) Colony formation of wildtype (*WT*) and *PD-L1* knockout (*KO*) MDA-MB-231 cells. Quantification of colonies in each group is shown in the left panel and PD-L1 western blot is shown in right panel. Data are presented as mean ± s.d., independently replicated three times. Statistical significance was calculated using 2-tailed Student's *t*-test. **P<0.01, ***P<0.001. (**b**) Colony formation of BT549 cells expressing control shRNA or two different PD-L1 shRNAs 14-16 days after cell seeding. Quantification of colonies in each group is shown in the left panel and knockdown efficiency is shown in the right panel. Data are presented as mean ± s.e.m., independently replicated three times. Statistical significance was calculated using Student's *t*-test. ***P < 0.001 (**c**) Protein levels of PD-L1, Sororin, and PD1 in breast cancer cell lines of different subtypes. Longer exposure time was used for PD1 blotting due to low protein level in breast cancer cells. (**d-f**) Colony formation of MCF7, ZR-75-1, and BT474 cells expressing control shRNA or two different PD-L1 shRNAs. (**g**) Protein levels of PD-L1 and Sororin in lung cancer, colon cancer and prostate cancer cell lines. (**h**) Colony formation of wildtype (WT) and *PD-L1* knockout (KO) RKO, HCC827, and DU145 cells. Data are presented as mean ± s.d., independently replicated three times. ***P<0.001, Student's *t*-test. (**i**) Colony formation of BT549 cells expressing control shRNA or two different PD1 shRNAs. Quantification of colonies in each group is shown in the middle panel and knock down efficiency is shown in the right panel. (**j-k**) MDA-MB-231 or BT549 cells were incubated with anti-PD-L1 monoclonal antibody (5 μg/mL and 10 μg/mL) or corresponding IgG isotype control for 6 days and the cell proliferation was analyzed at indicated time points. Data are presented as mean ± s.e.m., independently replicated three times. (**l**) Cytoplasmic/membrane (C/M) and nuclear (N) fractions of PD-L1 from MDA-MB-231 cells treated with either 5 μg/mL IgG or PD-L1 antibody were examined.
