# Supplementary material for: Regulation of sister chromatid cohesion by nuclear PD-L1
Source: Cell Res. 2020 Apr 29;30(7):590–601. doi: 10.1038/s41422-020-0315-8 (PMC7343880; doi:10.1038/s41422-020-0315-8)
Supplement: Supplementary file 2 — Supplementary FigS2 [file 41422_2020_315_MOESM2_ESM.pdf]

Supplementary Information, Fig. S2.

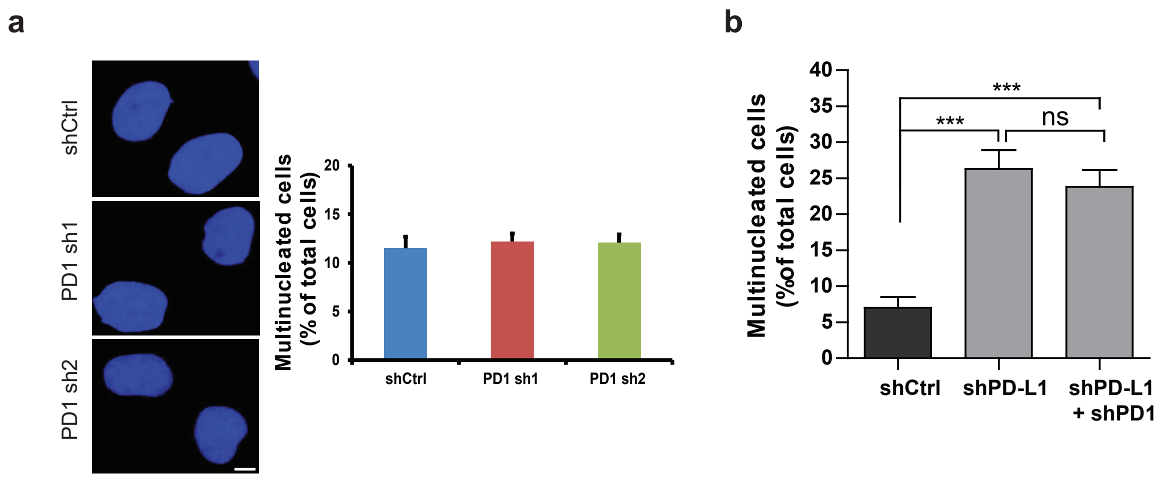

Supplementary Information, Fig. S2. Loss of PD-L1 but not PD1 induces formation of multinucleated cells.

(**a**) Three days after infection with control or PD1 shRNAs, cells were plated on cover slips and fixed. The cell nucleus was visualized by staining with DAPI. Bar: 10 µm. Multinucleated cells were quantified. (**b**) Quantification of multinucleated cells in cells expressing control shRNA, PD-L1 shRNA, or PD1 shRNA combined with PD-L1 shRNA. Data are presented as mean ± s.e.m., and were independently replicated three times with similar results. ***P<0.001, ns, not significant, Student's *t*-test.
