# Supplementary material for: Regulation of sister chromatid cohesion by nuclear PD-L1
Source: Cell Res. 2020 Apr 29;30(7):590–601. doi: 10.1038/s41422-020-0315-8 (PMC7343880; doi:10.1038/s41422-020-0315-8)
Supplement: Supplementary file 3 — Supplementary FigS3 [file 41422_2020_315_MOESM3_ESM.pdf]

Supplementary Information, Fig. S3.

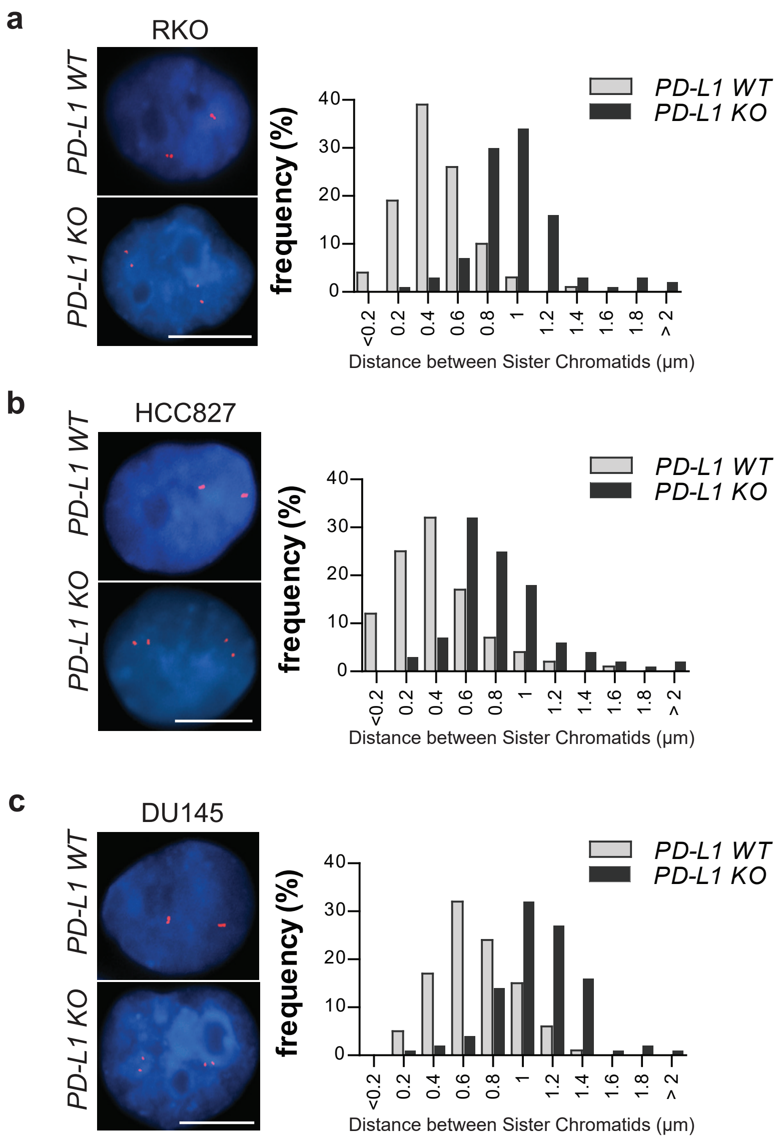

Supplementary Information, Fig. S3. PD-L1 regulates sister chromatid cohesion in different cancer cell lines.

(**a**-**c**) PD-L1 depletion impairs sister chromatid cohesion. *myb* gene distance between paired FISH signals in wildtype (WT) or *PD-L1* knockout (KO) RKO, HCC837, and DU145 cells. Bar: 10 μm.
