# Supplementary material for: Regulation of sister chromatid cohesion by nuclear PD-L1
Source: Cell Res. 2020 Apr 29;30(7):590–601. doi: 10.1038/s41422-020-0315-8 (PMC7343880; doi:10.1038/s41422-020-0315-8)
Supplement: Supplementary file 4 — Supplementary FigS4 [file 41422_2020_315_MOESM4_ESM.pdf]

Supplementary Information, Fig. S4.

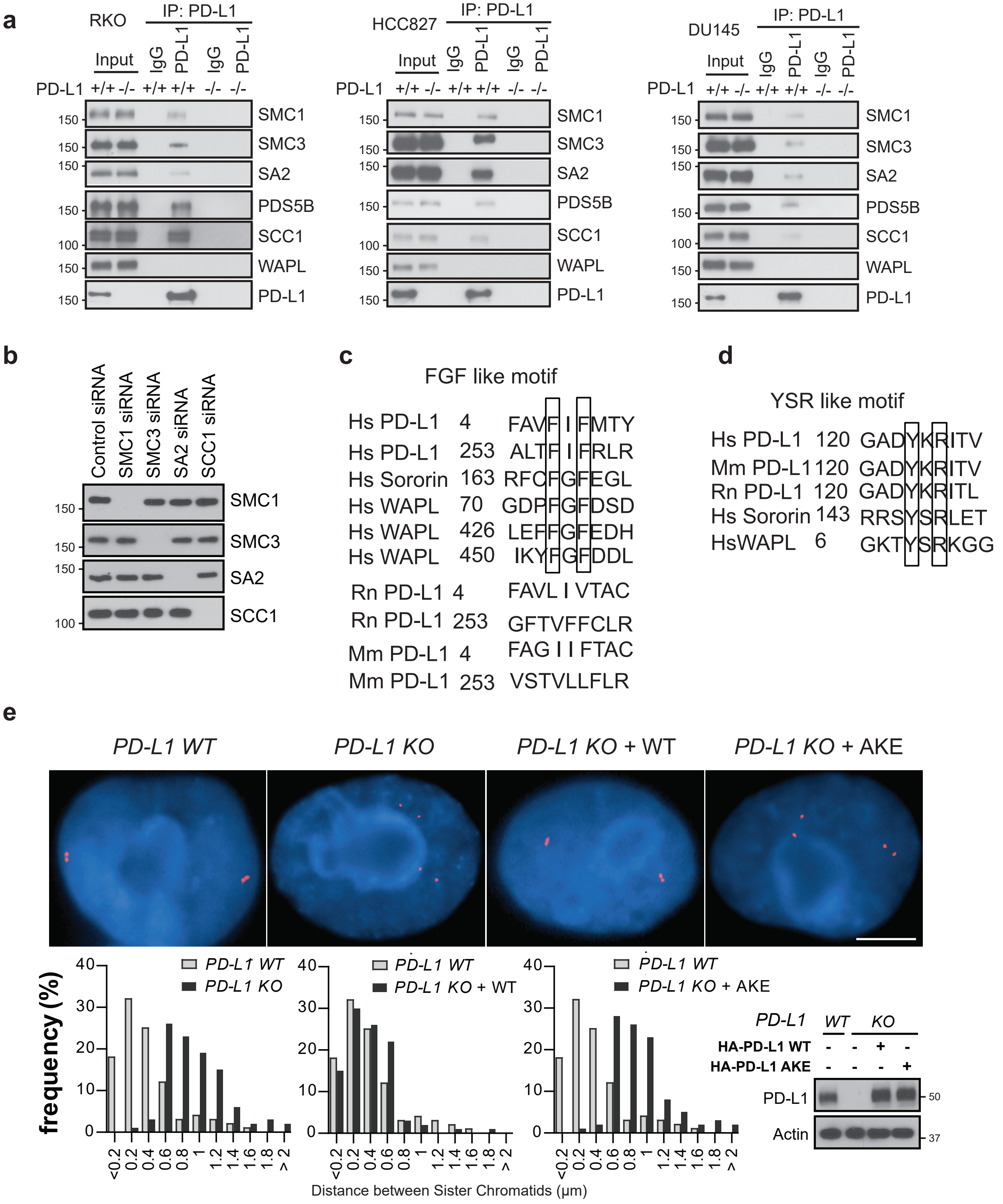

Supplementary Information, Fig. S4. PD-L1 interacts with the cohesin complex.

(**a**) Cohesin subunits can be co-immunoprecipitated with endogenous nuclear PD-L1 in RKO, HCC827 and DU 145 control (*WT*) cells, but not the knockout cells (KO). (**b**) The protein levels of individual cohesin subunits in the cell lysates isolated from cells transfected with siRNAs targeting individual cohesion subunit in the presence of MG132. (**c**) Analysis of FGF like motif in PD-L1 protein. (**d**) Analysis of YSR like motif in PD-L1 protein. (**e**) *PD-L1* knockout impairs sister chromatid cohesion; reintroduction of WT, but not the AKE mutant can rescue the defect in sister chromatid cohesion. *Myb* gene probe was used and the distance between paired FISH signals was measured and quantified. Bar: 5 μm. Data are presented as mean ± s.d., and were independently replicated three times.
