# Supplementary material for: Regulation of sister chromatid cohesion by nuclear PD-L1
Source: Cell Res. 2020 Apr 29;30(7):590–601. doi: 10.1038/s41422-020-0315-8 (PMC7343880; doi:10.1038/s41422-020-0315-8)
Supplement: Supplementary file 5 — Supplementary FigS5 [file 41422_2020_315_MOESM5_ESM.pdf]

Supplementary Information, Fig. S5.

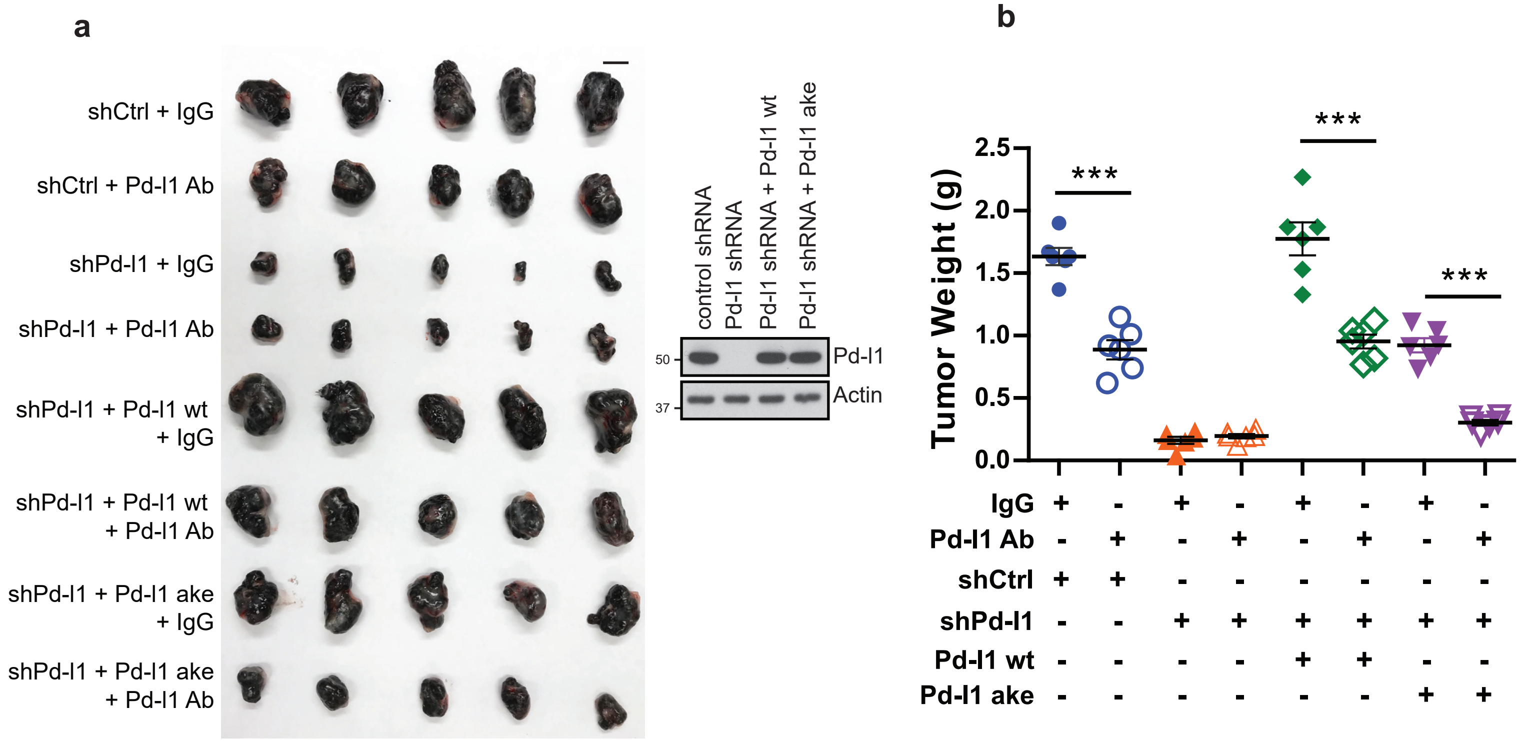

Supplementary Information, Fig. S5. PD-L1 AKE mutant impairs PD-L1 function in the nucleus while still maintaining its immunosuppressive effect.

(**a-b**) B16F10 cells expressing control shRNA (shCtrl), mouse Pd-l1 shRNA (shPd-l1), Pd-l1 shRNA plus shRNA resistant wildtype Pd-l1 (shPd-l1 + Pd-l1 wt), or mouse Pd-l1 shRNA plus shRNA resistant ake mutant Pd-l1 (shPd-l1 kd + Pd-l1 ake) were injected into C57/BL6 mice, and treated with 5 mg/kg mouse Pd-l1 blocking antibody or corresponding IgG. B16F10 xenograft tumors and tumor weight at the time of experiment termination are shown. Data are represented as the mean ± s.e.m. Dots depict individual data points. Statistical significance was calculated using Student's *t*-test. ***P<0.001.
