# Supplementary material for: Regulation of sister chromatid cohesion by nuclear PD-L1
Source: Cell Res. 2020 Apr 29;30(7):590–601. doi: 10.1038/s41422-020-0315-8 (PMC7343880; doi:10.1038/s41422-020-0315-8)
Supplement: Supplementary file 6 — Supplementary FigS6 [file 41422_2020_315_MOESM6_ESM.pdf]

Supplementary Information, Fig. S6.

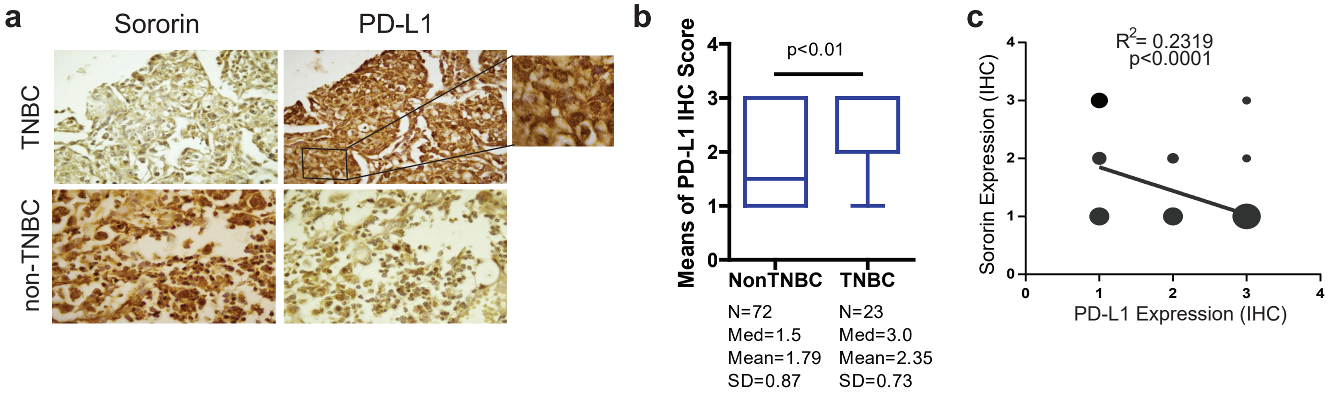

Supplementary Information, Fig. S6. PD-L1 negatively correlated with Sororin in TNBC patient tumors.

(**a**) Representative Immunohistochemistry (IHC) staining of PD-L1 and Sororin in the same region of a human breast cancer TMA. (**b**) Quantification of PD-L1 expression in non TNBC or TNBC primary breast cancer tissues. Box shows 25th-75th percentiles, as well as median (center line). P<0.01 by Student's *t*-test. (**c**) The correlation analysis of IHC scores (1, 2 and 3) of PD-L1 and Sororin using linear regression analysis. Dot size indicates the number of cases in this group.
