# Supplementary material for: Regulation of sister chromatid cohesion by nuclear PD-L1
Source: Cell Res. 2020 Apr 29;30(7):590–601. doi: 10.1038/s41422-020-0315-8 (PMC7343880; doi:10.1038/s41422-020-0315-8)
Supplement: Supplementary file 7 — Supplementary FigS7 [file 41422_2020_315_MOESM7_ESM.pdf]

Supplementary Information, Fig. S7.

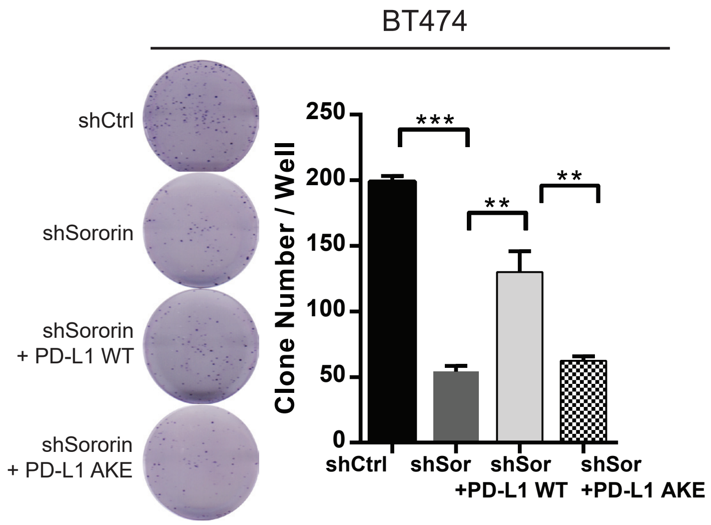

Supplementary Information, Fig. S7. PD-L1 compensates for the loss of Sororin and regulates sister chromatid cohesion.

Colony formation of BT474 cells expressing the indicated constructs. The experiment was repeated 3 independent times. **P<0.01, ***P<0.001 by Student's *t*-test.
